# Mesenchymal stromal cells as treatment for acute respiratory distress syndrome. Case Reports following hematopoietic cell transplantation and a review

**DOI:** 10.3389/fimmu.2022.963445

**Published:** 2022-11-08

**Authors:** Behnam Sadeghi, Olle Ringdén, Britt Gustafsson, Markus Castegren

**Affiliations:** ^1^ Translational Cell Therapy Research (TCR), Division of Paediatrics, Department of Clinical Science, Intervention and Technology, Karolinska Institutet, Stockholm, Sweden; ^2^ Department of Women’s and Children’s Health, Karolinska Institutet, Stockholm, Sweden; ^3^ Center for Clinical Research, Sörmland, Uppsala University, Uppsala, Sweden; ^4^ Department of Anesthesiology and Intensive Care, CLINTEC, Karolinska Institutet, Stockholm, Sweden; ^5^ Section of Infectious Diseases, Department of Medical Science, Uppsala University, Uppsala, Sweden

**Keywords:** mesenchymal stromal cells (MSCs), decidua stromal cells (DSCs), hematopoietic cell transplantation, acute respiratory distress syndrome (ARDS), cell therapy

## Abstract

Acute respiratory distress syndrome (ARDS) is a life-threatening lung disease. It may occur during the pancytopenia phase following allogeneic hematopoietic cell transplantation (HCT). ARDS is rare following HCT. Mesenchymal stromal cells (MSCs) have strong anti-inflammatory effect and first home to the lung following intravenous infusion. MSCs are safe to infuse and have almost no side effects. During the Covid-19 pandemic many patients died from ARDS. Subsequently MSCs were evaluated as a therapy for Covid-19 induced ARDS. We report three patients, who were treated with MSCs for ARDS following HCT. Two were treated with MSCs derived from the bone marrow (BM). The third patient was treated with MSCs obtained from the placenta, so-called decidua stromal cells (DSCs). In the first patient, the pulmonary infiltrates cleared after infusion of BM-MSCs, but he died from multiorgan failure. The second patient treated with BM-MSCs died of aspergillus infection. The patient treated with DSCs had a dramatic response and survived. He is alive after 7 years with a Karnofsky score of 100%. We also reviewed experimental and clinical studies using MSCs or DSCs for ARDS. Several positive reports are using MSCs for sepsis and ARDS in experimental animals. In man, two prospective randomized placebo-controlled studies used adipose and BM-MSCs, respectively. No difference in outcome was seen compared to placebo. Some pilot studies used MSCs for Covid-19 ARDS. Positive results were achieved using umbilical cord and DSCs however, optimal source of MSCs remains to be elucidated using randomized trials.

## Introduction

Acute respiratory distress syndrome (ARDS) is a life-threatening lung condition with a mortality rate that ranges between 25-50% (1–4). ARDS is characterized by acute hypoxic respiratory failure with bilateral pulmonary infiltrates that may be caused by septicemia, pneumonia, shock, following aspiration of gastric contents, or severe trauma (3, 4). ARDS may independently be caused by blood transfusion (5). ARDS is characterized by inflammation, neutrophil accumulation, a systemic and local inflammatory reaction, and alveolar injury. The damage induces protein-rich pulmonary edema, which leads to hypoxemia and impaired carbon dioxide (CO_2_) elimination. ARDS is classified as mild, moderate, and severe, based on the degree of hypoxemia. ARDS is used if the patient´s PaO_2_/FiO_2_ was less than 300 mmHg (6).

Diffuse alveolar damage (DAD) leading to high permeability followed by pulmonary edema is considered the histopathologic hallmark of ARDS. It usually happens when the patient already is critically ill or has had significant injuries (7, 8). ARDS causes the deaths of approximately 75.000 people in the United States annually (2, 7). In the Scandinavian countries, there are almost 170 patients/million inhabitants/year of ARDS with a mortality rate of 40% (9). Apart from substantial mortality, ARDS is associated with high morbidity rate and high costs on health systems. Patients with ARDS need ventilator support and are treated in intensive care units (ICUs) which is cumbersome and costly. Mechanical ventilation is required to keep good oxygenation and adequate CO_2_ elimination but may also lead to lung injury by rupturing healthy alveoli and triggering a secondary inflammatory response (1, 10).

Standard therapy for ARDS includes mechanical ventilation and fluid management therapy (11). There is no specific or targeted therapy against the underlying pathophysiological process of ARDS shown to be beneficial. For instance, β-2 agonist treatment and simvastatin were used for ARDS with no beneficial effects (12, 13).

Infection with the novel coronavirus, SARS-CoV-2, causing the ongoing Covid-19-pandemic, can lead to massive pro-inflammatory responses contributing to the development of ARDS and a concomitant cytokine storm reaction (14). The cytokine storm was studied in more detail however there is no consensus on a detailed definition (15, 16). Moreover, coagulopathy in these patients could possibly worsen the clinical outcome (17). Several ongoing trials investigate immunomodulatory therapies in Covid-19, including *e.g.* interleukin-6 inhibitors.

Various pulmonary problems are common following allogeneic hematopoietic cell transplantation (HCT) and include idiopathic pneumonia, viral- bacterial and fungal pneumonia, obstructive bronchiolitis and alveolar hemorrhage *etc (* 18). ARDS is rare after HCT but is associated with almost 100% mortality.

The rationale to use mesenchymal stromal cells (MSCs) stems from the very potent cellular immunomodulatory and anti-inflammatory effects (19–21). When MSCs are infused intravenously, they first home to the lungs (22–25). A study in mice showed that around 80% of the MSCs were found in the lungs within a few minutes after injection (26). MSC may not migrate beyond the lungs after intravenous infusion (27). We were the first group to treat ARDS using MSCs in a patient in 2004 (28).

In this article, we describe three patients treated with MSCs for ARDS following HCT and summarizes the immunomodulatory and anti-inflammatory effects of MSCs. We also review the experimental and clinical experience of treating ARDS with MSCs and future perspectives.

## Literature overview: Experimental and clinical experience

### Mesenchymal stromal cells

MSCs were first described by Friedenstein et al. (29). They described an adherent, fibroblast-like cell population in the bone marrow that could regenerate rudiments of bone *in vivo* (30, 31). MSCs were found in all tissues of the body and have been estimated to be 1/10.000 nucleated cells (32–34). Because MSCs can differentiate into several cells of mesenchymal cell lineages including bone, cartilage, tendons, cardiomyocytes, bone cells, and fat, they have raised interest in regenerative medicine (35, 36). There is no specific marker for MSCs, but they stain positive for CD29, CD73, CD90, CD105 and CD166 (32, 33, 37). They are negative for hematopoietic markers CD34, CD45, and CD14. MSCs have immunomodulatory effects and inhibit T-cell alloreactivity induced in mixed lymphocyte cultures (MLC) (20). MSCs suppression of alloreactivity is independent of the HLA system. HLA compatibility between MSCs and target lymphocytes are not necessary for immunosuppression to occur. This suggests the possibility to use third-party MSCs (21). Furthermore, the infusion of MSCs in humans was shown to be safe (38). Skin allograft survival was prolonged in baboons (19). These findings inspired us to treat a 9-year-old boy, with life-threatening grade IV acute graft-versus-host disease (GVHD), with MSCs. He had a most dramatic response and MSCs were also used in a pilot study by us including 8 patients (39, 40). Subsequently, MSCs are used to treat a wide range of inflammatory and autoimmune disorders such as Crohn’s disease, ulcerous colitis, multiple sclerosis, and ARDS among others (41–44).

MSCs have multiple effects on the immune system. MSCs increase the number of both CD4+ and CD8+ regulatory T cells and IL-10 production (45–49). MSCs decrease markers for activated T cells, CD25, CD69 and CD38 (50, 51). Furthermore, dendritic cells decrease TNF-α and IL-12 when co-cultured with MSCs (46). MSCs also increase IL-10 secretion by LPS-stimulated dendritic cells, and CD4+ cells had decreased IL-5 secretion.

Type 1 helper T cells (Th1) IFN-γ production was significantly decreased by MSCs. Type 2 helper T cells (Th2) increased IL-4 secretion in the presence of MSCs. MSCs inhibition of alloreactivity in mixed lymphocyte cultures and subsequent development of cytotoxic T cells was induced by a soluble factor. Several immunoinhibitory soluble factors are produced by MSCs, among them are prostaglandin E2 (46), HLA-G5 (52). The immunoinhibitory effect by MSCs is augmented by IFN-γ (53, 54). MSCs can also be activated by stimulation of their Toll-like receptors (55). The T-cell inhibitory enzyme indoleamine-2,3 dioxygenase (IDO), is induced by IFN-γ, which catalyzes the conversion from tryptophan to kynurenine and inhibits T cell responses (56, 57). IDO reduced by MSCs is involved in the induction of regulatory T cells and the inhibition of T helper 17 (Th17) differentiation (58, 59). IDO-produced MSCs can also promote the differentiation of macrophages towards the M2 phenotype (60). MSCs have been reported to induce macrophage reprogramming through an anti-inflammatory/immunosuppressive profile (61, 62). MSCs may also inhibit reactivity by T cell inhibitory molecule PD-L1 (60). MSCs may use adenosine-mediated suppression *via* CD39 and CD73 to suppress activated T cells (63). Inhibition and apoptosis of T cells may also be mediated by galectin induced by MSCs (64). It has been proposed that MSCs decrease B cell proliferation when used in a high concentration of MSCs (65). In contrast, lower doses of MSCs stimulated B cell antibody secretion in human spleen cells (66). MSCs decreased anti-inflammatory gene 6 protein (TSG-6) also stimulated by human tumor necrosis factor α (TNF-α), to induce an anti-inflammatory effect (67). MSCs immunosuppressive activity also involves the engagement of regulatory T cells (48, 49).

Activated MSCs can modulate adaptive immune cells through contact-dependent mechanisms. It includes activation of the PD-1 pathway (60), FAS-mediated T cell apoptosis (68) engagement of VCAM-1 and ICAM-1 (69), and through upregulation of CD39 and increasing adenosine production (63). Direct contact is also important for MSCs induced immunosuppression in mice (70). In a murine model of GVHD, it was demonstrated that MSCs are actively induced to undergo perforin-dependent apoptosis by recipient cytotoxic T cells and that this process is essential to initiate MSCs-induced immunosuppression (71). After infusion recipient phagocytes engulf apoptotic MSCs and produce indoleamine 2,3-dioxinease, which is necessary for inducing immunosuppression. A recent study in NSG mice demonstrated that shortly after intravenous administration, MSC become apoptotic in the lungs and are eliminated locally by several phagocyte subsets. Efferocytosis by alveolar macrophages is critical for the therapeutic effect of MSC (72). In addition, administration of apoptotic human umbilical cord blood-derived MSC exerts a beneficial effect in acute lung injury in rats (73). The importance of MSCs apoptosis and efferocytosis for MSC immunosuppression was also found by de Witte et. al (74).

MSCs were thought to be immune-privileged cells (53, 75). However, in a baboon model, it was shown that alloantibodies were formed after infusion of allogeneic MSCs (76). Furthermore, MSCs are rejected in allogeneic mice (77). We did not detect any anti-HLA antibodies in GVHD patients treated with MSCs or DSCs in patients who have undergone HCT (78, 79). However, a small girl diagnosed with epidermolysis bullosa developed multi-specific anti-HLA antibodies after repeated infusion of DSCs.

After infusion in human blood, MSCs were susceptible to complement activation (80), which resulted in cell death (81). Coagulation factors are activated when MSCs are infused into the blood (82).

Exosomes and macrovesicles derived from MSCs were shown to protect from acute kidney injuries (83) myocardial ischemia (84), and pulmonary hypotension (85) in various animal models. MSC-derived exosomes have also been used in ARDS/ALI (86–88).

Infusion of MSCs seems safe and there are few side-effects reported (89). In a meta-analysis from more than 1000 patients who were treated for various disorders such as GVHD among others, the toxicity of allogeneic MSCs were limited to transient fever (90).

The most commonly used source of MSCs is the bone marrow (BM) from which it is renewable (38, 91). Adipose tissue is commonly used as a source of MSCs (92). Other sources of MSCs include the umbilical cord and placenta (34, 93, 94).

The most common indication for MSCs therapy in HCT patients is acute GVHD with numerous encouraging pilot studies (40, 95–98). Other indications following HCT are chronic GVHD, hemorrhagic cystitis, pneumomediastinum (99), and in a few patients reported here ARDS. The immunomodulatory capacity of MSCs has also been used to treat autoimmune inflammatory disorders such as multiple sclerosis (100). Other immune disorders treated by MSCs include rheumatoid arthritis (41), inflammatory bowel disorders (42), and Systemic Lupus Erythematosus (43). MSCs are used to repair several mesodermal tissues including bone, tendon, cartilage, and fat. They were also used to repair cardiac damage (44). Furthermore, they have been used to prevent aging (101).

### Experimental studies using mesenchymal stromal cells for sepsis and ARDS

There is no specific treatment for sepsis and ARDS and the therapy is mainly supportive. However, there is growing evidence of the potential of cell-based therapies for the treatment of sepsis and ARDS. Worldwide more than 5 million people die every year due to sepsis (102).

Symptomatic therapy for ARDS include low-tidal volume ventilation (103), neuromuscular blockage (104), prone position (105), and extracorporeal membrane oxygenation (106).

### Experimental studies

For a decade MSCs have been studied in several experimental models of sepsis and ARDS. In a mouse model, it was demonstrated that MSCs attenuated septicemia and the effect was dependent on MSCs-derived prostaglandin E_2_, which reprogrammed macrophages in septic lines to produce anti-inflammatory IL-10 (61). Similarly, MSCs also promote recovery and repair lung injury in rats (107, 108). In a lung contusion injury model followed by hemorrhagic shock in rats, MSCs decreased injury in part by increasing the proportion of regulatory T cells (109). Human MSCs also reduced bacteremia and mortality in mice with gram-negative sepsis (110). In this model, MSCs altered the polarization of alveolar macrophages to an M2-like anti-inflammatory phenotype. The phagocytic ability of macrophages was also enhanced by MSCs in mice who had pneumonia and peritoneal sepsis (111). Similar effects were also seen in a human lung injury model (112).

Devaney et al. also showed that human MSCs improved lung injury induced by *E.coli* in rats by decreasing levels of alveolar neutrophil inflammation and increased levels of IL-10 and subsequently enhanced function of macrophages (113).

In a mouse model of endotoxin-induced ALI, it was shown that intrapulmonary infusion of BM-MSCs improved survival and attenuated lung injury (114). In mice, it was shown that MSCs resolution of endotoxin-induced lung injury partly was mediated by lipoxin-A_4_, derived from arachidonic acid (115). Condor and colleagues demonstrated in a sepsis-induced multi-organ failure model that human MSCs increased production of prostaglandin E_2_ and IL-10 and improved survival (116). Several investigators showed in mice models that administration of human MSCs from various origins i.v. to mice, had positive effects on ALI such as decreased IL-1β, lung edema, decreased IL-1β in bronchoalveolar lavage (BAL), reduced inflammation, and enhanced anti-apoptotic effects (117). Similarly, human umbilical cord-derived MSCs (UC-MSCs) reduced ARDS in rats and improved survival (118). In most of these studies, lung injury was induced by lipopolysaccharide (LPS). Various strategies were also used to enhance the effects of MSCs in the treatment of experimental ARDS. In an LPS model of lung injury in mice, Wang et al. used MSCs overexpressing IL-10 which increased B- and T-cell production of IL-10 and decreased TNF-α, and improved survival (119). The toll-like receptor 3 ligands, poly (I:C), led to increased prostaglandin E2 production and enhanced effect by MSCs on macrophage function in a sepsis model (120). Methods to potentiate the effects of MSCs in experimental ARDS include overexpressing soluble ST2 *via* lentiviral transfection (121), MSCs transfused with the E-prostanoid 2 receptor (122), overexpression of receptor tyrosine kinase (ROR2) (123), overexpression of platelet-derived growth factor receptor (124), overexpression of ANG-1 (125), overexpression of angiotensin-converting enzyme-2 (126), and overexpression of the gene Angiotensin-converting enzyme growth factor-2 (127). The sphingosine-1 phosphate analog FTY720 was affected together with human MSCs in ARDS in mice (128). Exosomes from MSCs were also beneficial to treat ARDS in mice (88). However, a study showed a better response of lung injury using cells as compared to secretomes (129).

### ARDS following hematopoietic cell transplantation

Pulmonary complications are common in patients, following HCT (18, 130, 131). Pulmonary disorders following HCT include, idiopathic pneumonia syndrome, interstitial pneumonitis, diffuse alveolar hemorrhage, peri-engraftment ARDS, noncardiogenic capillary leak syndrome, bronchiolitis obliterans and broncho-pneumonia. ARDS is not so common, but a severe condition post HCT. In a study including 3920 HCT patients, 4,5% developed ARDS, with a one-year mortality of 70% (132). At the end stage of respiratory distress syndrome, the patients may present with hypoxic respiratory failure, meeting the full criteria for ARDS according to the Berlin definition. Among 2635 patients following allogeneic or autologous HCT, 133 cases 5,0% developed ARDS (133). One year mortality was 67%.

Patients who develop ARDS following HCT have a very poor outcome. In the 1990th mortality approached 100% in the intubated patients (134). In intubated patients with multiple organ failure (MOF) after HCT, mortality was 94-100%. With such a dismal outlook, palliative care was commonly chosen, in those days.

Improvements in intensive care in more recent years, as using ventilation in a prone position, restrictive fluid therapy, new diagnostic methods, and possibility to treat pathogens, have resulted in improved survival rate in this group. Extracorporeal membrane oxygenation (ECMO) was used to treat ARDS in 37 adult patients at a median of 147 days after HCT (135). Overall, 7(19%) of the patients survived until discharge. Patients treated more than 240 days after HCT had a better survival 6/13(46%) as opposed to 1/24(4%) among those treated early(p<0.001). In pediatric patients with ARDS, survival was reported to be 24,6% (136). MSCs were often used to treat severe acute GVHD following HCT (98). However, MSCs were rarely used to treat ARDS after HCT. Therefore, these case reports are of interest.

### Clinical experience using mesenchymal stromal cells for ARDS

There is so far limited experience using MSCs in patients compared to the multiple studies in experimental models of ALI/ARDS. Adipose-derived MSCs were used in a small prospective randomized placebo-controlled study including 12 patients with ARDS (137). Intravenous Adipose-MSCs 1x10^6^ cells/kg vs placebo was given and there was no difference in outcome in patients treated with Adipose-MSCs versus placebo. In a phase I study, 3 patients each with ARDS received 1x10^6^, 5x10^6,^ or 10x10^6^ MSCs/kg as a single intravenous dose for ARDS (138). There were no MSC infusion-related hemodynamic, or respiratory adverse events reported. The treatment was well tolerated and the dose of 10x10^6^ cells/kg was selected for further study. A randomized phase 2 safety trial using 10x10^6^ BM-MSC/kg showed no statistical difference in survival between the BM-MSC groups (n=40) and the placebo group (n=20) (139),. The primary endpoint was safety, and it was shown that it was safe to infuse 10x10^6^ BM-MSC/kg in patients with moderate to severe ARDS. There were no MSCs infusion-related hemodynamic, or respiratory adverse events reported. In a separate study it was found that compared with placebo, MSC treatment significantly reduced airspace total protein, angiopoietin-2 (Ang-2), IL-6, and soluble TNF receptor-1 concentrations (140). In conclusion from these randomized studies in ARDS patients, adipose or BM-MSCs were safe to infuse, but did not improve survival compared to placebo.

### Mesenchymal stromal cells for COVID-19-induced ARDS

Several trials are registered to use MSCs to treat Covid-19-induced ARDS (141). A study showed that MSCs are resistant to SARS-CoV-2-infection and retain their immunomodulation potential, supporting their applicability for Covid-19-induced ARDS (142). Five patients were given 1x10^6^ UC-MSCs/kg for Covid-19 ARDS. After infusion of UC-MSCs, there was a rise of PaO_2_/FiO_2_ in all patients, three patients survived. In a German study 5 out of 23 patients with severe Covid-19 were treated with 1x10^6^ MSCs/kg (143). Four of the five BM-MSC-treated patients got ECMO to support vs 9/18 of the controls. Four of the 5 BM-MSCs-treated patients survived to discharge compared to 8/18 of the controls. A study used UC-MSCs in six and placenta-derived MSCs in 5 patients (144). The patients received fixed doses, 200x10^6^ MSCs infused every other day for 3 doses. No side effects were seen and 6 of the 11 patients survived (144). UC-MSCs were used in a double-blind pilot study including 24 Covid-19 ARDS patients (145). Two fixed doses of 100x10^6^ UC-MSCs were given to 12 patients. UC-MSCs infusions were safe. Survival was 91% in the UC-MSC group compared to 42% among the controls (p=0.015) (145). A Chinese study compared 26 patients treated for Covid-19 for ARDS with 3 doses of MSCs from menstrual blood with 18 control patients (146). Safety was the same between the two groups. Mortality was 7.69% in the MSCs group as opposed to 33.33% among the controls (p=0.048). During the Covid-19 pandemic several groups conducted small phase 1 trials using MSCs (147–154). Monsel et al. did not find an improved outcome following 3 infusions of UC-MSC compared to the control group (151). Dilogo et al. randomized 20 patients to UC-MSC and 20 patients to placebo. 10 patients in the UC arm survived compared to 4 patients in the placebo arm (P<0.05). Length of stay in ICU and ventilator usage did not differ between the two groups (153). In a prospective randomized study, enrolling 101 patients, who were given UC-MSCs or placebo, the UC-MSC group, the UC-MSC group had a trend for numerically improvement in whole lung lesion volume on day 28 (P<0.08) (152). Gregoire and colleagues used BM-MSCs in a phase I/II trial and treated 8 intensive care patients with Covid-19 induced ARDS with three i.v. infusions at three-day intervals with 1.5-3x10-6 cells/kg (155). All MSCs treated patients were alive at 60 days which was significantly higher compared to 70.8% in 24 well-matched control patients, who were not given MSCs (p<0.0082).

DSCs were given to l0 ARDS patients with Covid-19 disease in Tehran (156). Pulmonary infiltrates disappeared in all patients. Seven patients survived and were discharged. To conclude, several trials used MSCs from different origin to treat Covid-19 induced ARDS. Some promising outcomes were seen using UC-MSCs and DSCs.

## Materials and methods

### Patients

Following HCT three patients developed ALI/ARDS and were treated with BM-MSCs in two cases and DSCs in the third case (28). This is a case study, conducted in an academic setting. ARDS is defined according to the Berlin criteria (157). ALI have similar clinical features without fulfilling all ARDS criteria according to the Berlin definition (158).

The first patient was a 22-year-old male with acute lymphoblastic leukemia in second remission, who was given a cord blood graft (**Table 1**). The second patient was a 13-year-old boy with acute lymphoblastic leukemia (ALL) with a graft from an unrelated donor. A 33-year-old man with chronic myeloid leukemia, resistant to Nilotinib, received a graft from an HLA-identical unrelated donor (**Table 1**). The first two patients were conditioned before transplant with cyclophosphamide and fractionated total body irradiation (159). The third patient was conditioned with cyclophosphamide and busulfan. Prophylaxis against GVHD was methotrexate and cyclosporine. The transplantation procedure was previously published in detail (160, 161) (**Table 1**).

**Table 1:** Characteristics of three patients, diagnosed with ARDS.

	Patient 1	Patient 2	Patient 3
**Age**	22	13	32
**Sex**	M	M	M
**Diagnose**	ALL, 2CR	ALL, 2CR	CML
**HLA donor (match)**	MMUD	MUD	MUD
**Conditioning**	Cy/fTBI	Cy/fTBI	Bu/Cy
**ATG**	+	+	+
**Immunosuppression**	CyA + Pred.	CyA + MTX	CyA + MTX
**Graft source**	2 CB	PBSC	PBSC

M, male; ALL, acute lymphoblastic leukemia; CR, complete remission; CML, chronic myeloid leukemia; MMUD, mismatched unrelated donor; MUD, matched unrelated donor; Cy, Cyclophosphamide; fTBI, fractionated total body irradiation; Bu, busulfan; ATG, anti-thymocyte globulin; CyA, cyclosporine; MTX, methotrexate; CB, cord blood; PBSC, peripheral blood stem cells. + means that the patients received ATG.

### Cell culture

The culture method and expansion of MSCs and DSCs was previously published in detail (21, 162). The stromal cells expressed CD166, CD105, CD73, CD44, and CD29 but not hematopoietic markers CD34, CD14, and CD45. The cells were cultured, and control samplings were negative for bacteria, mycoplasma, and fungi during expansion and before infusion. The stromal cells were cultured and expanded in a good manufacturing process condition, at the Department of Clinical Immunology, Karolinska University Hospital, Huddinge, Sweden.

Initially we used BM-MSCs for immunomodulation in HCT patients (40, 99). Due to insufficient clinical effect using BM-MSCs and a better immunomodulatory effect by DSCs, compare to BM-MSCs, we later switched to DSCs (94, 163, 164).

The MSCs and DSCs were stored in liquid nitrogen, thawed, and suspended in cliniMACS PBS/EDTA buffer, supplemented with 10% AB plasma for MSCs and 5% albumin for DSCs. The cells were washed three times and resuspended in NaCl and 10% AB-serum (MSCs) or 5% albumin (DSCs) (164, 165). The infusion solution was then filtered through a 70μM cell strainer (BD Bioscience, Franklin Lakrs, NI). It was then transferred to a heparinized syringe at 2x10^6^ cells/ml. The MSCs and DSCs were infused i.v. *via* a central venous line. Dosing aimed at 1x10^6^ MSCs/kg, due to the number of cells in frozen vials and following thawing, the final dose given to the three patients varied from 1.0-1.4 x10^6^ cell/kg in this report. The line was flushed with 2-5 ml NaCl containing 50 IE heparin/ml in the two adults and 25 IE heparin/ml in the boy.

## Results

Patient 1 underwent HCT in 2004. Following initial acute gastrointestinal GVHD with diarrhea, he developed hemorrhagic cystitis and was treated with 0.7x10^6^ MSCs/kg on day 81 after HCT. A week before MSCs infusion he required 8 units of erythrocyte concentrates, 14 units of fresh frozen plasma, and 9 platelet transfusions. The week following MSCs infusion he only required 2 platelet transfusions and no erythrocyte transfusions. On day +93 he worsened and developed multiorgan failure. Pulmonary X-ray revealed ALI/ARDS. On day +96 he was given a second dose of 1.4x10^6^/kg of HLA haploidentical MSCs from his mother (**Table 2**). Following this, a new X-ray was performed and showed clear lungs with no signs of ALI/ARDS (**Table 2**). Despite this, the patient deteriorated and died on day +104 due to multi-organ failure. Patient developed renal, liver, and cardiac failure, which was the cause of death (**Table 2**). Autopsy revealed that the bladder was healed.

**Table 2:** Outcome of three patents, diagnosed with ARDS and treated with MSCs.

	Patient 1	Patient 2	Patient 3
**ARDS ethology**	aGVHD, HC (4), Multiple transfusion	Granulocyte transfusion	Alfa streptococcal septicemia
**MSCs source**	BM	BM	DSCs
**MSC donor**	Haploidentical	Third party	Third party
**Cell dose x10^6^ MSC/kg**	1.4	1.0	1.0
**Effect on ARDS**	Lungs cleared	Progression	Lungs cleared
**Verification by**	X-ray	X-ray/ventilatory support needed	X-ray/discontinued oxygen supply
**Outcome**	Death due to liver, kidney dysfunction; finally, cardiac arrest	Death due to aspergillus pneumonia	Recovered, alive and well (for more than 7 years)

ARDS, acute respiratory distress syndrome; aGVHD, acute graft versus host disease; HC, hemorrhagic cystitis [grade 4= life threatening (1)]; MSCs, mesenchymal stromal cells; BM, bone marrow; DSCs, decidua stromal cells.

Patient 2 was a thirteen-year-old boy with ALL and prolonged neutropenia after HCT. Due to this, he was treated with granulocyte transfusions to treat his bacterial infection. Following this, he had deteriorated pulmonary function and an X-ray showed bilateral pulmonary infiltrates (**Table 2**). This was thought to be due to granulocyte transfusion. Due to the picture of ALI/ARDS, he was treated with 1x10^6^ MSCs/kg. Following this, he required ventilatory assistance and died of massive aspergillosis pneumonia as found at autopsy (**Table 2**). The reason of death was pulmonary failure. The role of BM-MSCs in exacerbating invasive fungal infection is discussed in the discussion (second paragraph).

Patient 3 was a thirty-three-year-old man with chronic myeloid leukemia who underwent a transplant in 2014 and developed septicemia during the neutropenic phase after HCT. On day +8 he had a fever (40°C) and was treated with high doses of piperacillin/tazobactam. Chest radiography was normal. However, the fever continued, and the maximum pulse increased to 155/min. Blood culture showed alpha streptococci. Hemoglobin decreased from 100 to 60g/l. Weight increased by 5 kg. On day +10 he was short of breath and chest radiography suggested ARDS (**Table 2**). He required oxygen supply, and this was increased to 15L/min. He was given large doses of albumin and furosemide. Oxygen saturation varied between 92-96%. The patient was completely exhausted and needed ventilatory support. From a compassionate basis and according to the Declaration of Helsinki, he was given 1x10^6^ DSCs/kg on day +11. Oxygen saturation instantly increased from 88-92% to 98% (and was thereby stabilized). Oxygen supply, which was successively decreased, was discontinued five days later. Chest radiography improved and was normal on day +22 after HCT when the patient was discharged from the hospital (**Table 2**). He had elevated granulocyte colony-stimulating factor, IL-6, IL-8, and MCP-1 which all decreased 3 hours after DSC infusion and normalized one week after infusion. The patient is alive and well after 7 years.

## Discussion

The reason we used MSCs for the treatment of ARDS was that these cells first home in the lungs when given i.v (22, 24, 25). and they have a very potent immunomodulatory and anti-inflammatory effect (19, 20, 166). The hope was that they would turn off the ARDS-induced cytokine storm in the lungs. Recently Wick KD et al. showed that MSCs infusion locally decrease the inflammatory cytokine level compared to the control group (140). In our first patient, the pulmonary infiltrates cleared (**Table 2**). However, he suffered from multiple medical problems and finally died from multi-organ failure.

The second patient died from invasive aspergillosis. An invasive fungal infection may be a problem after MSC therapy (163). This could be due to local immunosuppression following MSCs infusion, paving the way for invasive fungal infection (140). We also experienced an increased risk of invasive fungal infection and death by pneumonia in HCT patients given MSCs (167). Therefore, antifungal prophylaxis should be considered in patients treated with MSC following HCT (28).

The third patient responded dramatically to DSCs with a very rapid recovery. Reasons for this better response compared to BM-MSCs may be the more potent immunosuppressive and anti-inflammatory effects by DSCs as opposed to BM-MSCs seen *in vitro* and *in vivo* for the treatment of acute GVHD (94, 164). Although BM-MSCs and DSCs have similar surface markers, such as CD105, CD166, CD73, CD90, and CD29, there are several differences between BM-MSCs and DSCs apart from a more potent immunosuppressive effect by DSCs (162, 168). DSCs are half the size of BM-MSCs and do not differentiate well to fat and cartilage (169).

In the ARDS patient who was successfully treated by DSCs, cytokines/chemokines G-CSF, IL-6, IL-8, MCP1, and TNF-α all decreased dramatically in blood. In addition, DSCs also decrease IFN-γ and IL-17 (94). All these cytokines contribute to the cytokine storm during ARDS (16, 170).

Furthermore, DSCs compared to BM-MSCs, have a stronger expression of CD49b, a marker for homing to inflamed tissue. This may be of importance in the context of ARDS. The data presented here must of course be taken with a lot of caution because these are only three anecdotal cases and performed over ten years. In fact, ARDS is relatively rare following HCT. The more promising immunomodulatory and anti-inflammatory response using DSCs as opposed to BM-MSCs includes also patients treated for acute GVHD (n=38) and hemorrhagic cystitis (n=11) (164, 171). Although these studies suggest that DSCs have a better immunosuppressive effect as opposed to BM-MSCs (164), well-designed prospective randomized trials are necessary to prove the superior efficacy of DSCs.

In the literature, numerous preclinical studies are supporting the effects and rationale to use MSCs for sepsis and ARDS, some of which are referred to in this review, and there are more published (172–174). Concerning GVHD, MSCs from different sources do not work very well to reverse this disorder in mice in contrast to humans (175).

In mice BM-MSCs need to be licensed by various methods, like treatment with IFN-γ, insertion of IL-10 promoting genes, and more, to be effective to reverse acute GVHD. In a similar way, several methods to enhance the anti-inflammatory effect of MSCs were also used for ARDS and sepsis in several animal models. Several of these modalities are previously described in detail (176).

In the clinic and for manufacturing of MSCs, it may be somewhat more problematic if gene-manipulation is needed to make MSCs useful for therapy.

In the limited clinical experience using MSCs, the two randomized ARDS studies so far, did not show any difference in outcome comparing MSCs from adipose or BM and placebo (137, 139). Although there were no significant differences in survival between the MSC patients and the placebo controls, there was a numerically increased mortality in the patients in the MSC group. This was due to the fact that the patients in the MSC group had numerically higher severity scores. A concern with this trial may be that viability of the MSC at infusion was rather poor 36%-85%. In our clinically responding DSC-treated patients, DSC viability was a median of 95% (164). Although it has been shown that MSCs immunosuppressive function in the body comes after apoptosis (71, 72), but it is important that we inject live cells rather than dead cells. When the cell dies outside the body, they might not have the same apoptotic or efferocytosis effects *in vivo*. The high numbers of BM-MSC 10x10^6^/kg compared to 1x10^6^ DSC/kg in our DSC-treated patient did not seem to compensate for poor cell viability.

In all clinical studies so far, it appears safe to inject MSCs and there were no serious adverse events reported. This is also in line with two meta-analyses regarding the safety of MSC therapy (89, 90). MSCs appear safe and side-effects seem to be limited to a slight fever. There is much less experience with DSCs. However, safety data in rats and mice as well as from the clinic suggest that they are as safe to inject as MSCs from BM (165, 177). In the clinical experience, side-effects were limited to three cases at the time of infusion and were spontaneously reversible without the need for any medical intervention.

Even if there is limited clinical experience using MSC to treat ALI/ARDS, it is worth pursuing this potentially effective therapy. As stated in this review and some other reviews, MSC therapy is demonstrated to be effective in several preclinical models of sepsis and ARDS (108, 172–174). In the clinic, several questions need to be addressed before we have an optimal stromal cell therapy for ARDS. What is the optimal source, bone marrow, adipose, UC, or placenta-derived? What is the optimal dose, and what is the best route of administration, i.v. or intratracheal? Intramuscular is probably less effective for ARDS because you want to shut off the cytokine storm in the lung. Are soluble factors and exosomes more or less useful than stromal cells (178)? Will priming with e.g., IFN-γ, TNF-α, IL-1α or -β or IL-17A be beneficial (176)? There are several unanswered questions to be studied before this promising therapy can move forward and be an established therapy for sepsis and ARDS. Technically allogeneic MSCs should be used for ARDS, because they can be prepared and taken “off the shelf”. Autologous MSCs in not an option because it takes several weeks to expand them to a meaningful number for clinical use.

With the global threats of the new coronavirus, Covid-19 pandemic, mortality noted in 3.7% of the infected patients seems to be due to ARDS in most (88%) of the patients (179). Many of the COVID-19 patients are in a hypercoagulable procoagulant state and at high risk for disseminated intravascular coagulation, thromboembolism, and thrombotic multi-organ failure (17). When choosing a therapeutic approach with or without cell based therapy we should consider this condition.

Therefore, it is an urgent need for effective therapy of ARDS. The burden of ARDS patients needing ventilator assistance is tremendous, and hopefully, stromal cell therapy may be used successfully to limit the burden on intensive care units worldwide.

In ARDS induced by bacterial sepsis, there are most effective antibiotics to be used, like in our HCT case treated for ARDS by DSCs. For Covid-19, there is no effective drug to inhibit viral replication and subsequent dissemination to the heart, kidneys, and intestine. In some cases, it may be sufficient to turn off the cytokine storm and the alveolar damage. In others, this may not be enough. There is limited clinical experience using various sources of MSCs for Covid-19-induced ARDS. So far, MSCs from UC and DCSs hold some promise (145, 156).

In conclusion, MSCs from various tissues, like BM, adipose, UC, and placental tissues, have taken the step from experimental animals to clinical trials. We have added three rare HCT cases to the so far limited clinical experience. One of them had a most dramatic clinical response to DSCs treatment. There are too few HCT patients with ARDS, so large prospective studies have to be performed in other patients’ groups. There are increasing numbers of pilot studies reporting on the use of various sources of MSCs for Covid-19-induced ARDS. Well-designed prospective placebo-controlled studies are needed to prove efficacy.

## Ethics statement

We had ethical approval to harvest MSCs from volunteer donors and to treat the patients against GVHD and other toxicities following HCT (Dnr 328/03). We also had ethical permission to harvest DSCs from Caesarean section placentas and use them for the treatment of GVHD and other toxic conditions after HCT (2009/418-31-34 and 2010/2061-32, 2010/452-31/4 and 2014/2132-32). The procedure to use DSCs was also approved by the Central Ethical Review Board in Sweden (Dnr 011-2016). Bone marrow (BM) was aspirated from volunteer donors. The methods for clinical culture of DSCs were previously approved by the Swedish Product Agency (Dnr 6.1.3 – 42994/2013). Written informed consent was obtained from the individuals and, regarding the pediatric patient, both parents gave informed consent for the publication of any potentially identifiable images or data included in this article.

## Author contributions

OR wrote the first draft of the article, complemented, and revised by BS, BG, and MC. All authors contributed to the article and approved the submitted version.

## Funding

The study was supported with grants from the Swedish Cancer Foundation (CAN 2018, 419) and the Cancer Society in Stockholm (111293). OR was the recipient of a Distinguished Professor Award from the Karolinska Institutet. BG was recipient of grant (PR2020-0058) from Swedish children cancer society (Barncancerfonden).

## Acknowledgments

We thank Gunilla Tillinger for the excellent typing of this manuscript.

## Conflict of interest

The authors declare that the research was conducted in the absence of any commercial or financial relationships that could be construed as a potential conflict of interest.

## Publisher’s note

All claims expressed in this article are solely those of the authors and do not necessarily represent those of their affiliated organizations, or those of the publisher, the editors and the reviewers. Any product that may be evaluated in this article, or claim that may be made by its manufacturer, is not guaranteed or endorsed by the publisher.
